# Cell activation and HIV-1 replication in unstimulated CD4^+^ T lymphocytes ingesting exosomes from cells expressing defective HIV-1

**DOI:** 10.1186/1742-4690-11-46

**Published:** 2014-06-12

**Authors:** Claudia Arenaccio, Chiara Chiozzini, Sandra Columba-Cabezas, Francesco Manfredi, Maurizio Federico

**Affiliations:** 1 National AIDS Center, Istituto Superiore di Sanità, Viale Regina Elena, 299, Rome 00161, Italy; 2 Department of Science, University Roma Tre, Rome, Italy; 3 Department of Cell Biology and Neurosciences, Istituto Superiore di Sanità, Rome, Italy

**Keywords:** Defective HIV-1, Exosomes, ADAM17, CD4^+^ T lymphocytes, Nef

## Abstract

**Background:**

A relevant burden of defective HIV-1 genomes populates PBMCs from HIV-1 infected patients, especially during HAART treatment. These viral genomes, although unable to codify for infectious viral particles, can express viral proteins which may affect functions of host cells as well as bystander ones. Cells expressing defective HIV-1 have a lifespan longer than that of cells producing infectious particles. Hence, their interaction with other cell types, including resting lymphocytes, is expected to occur frequently in tissues where HIV actively replicates. We investigated the effects of the expression of a prototype of functionally defective HIV-1 on bystander, unstimulated CD4^+^ T lymphocytes.

**Results:**

We observed that unstimulated human primary CD4^+^ T lymphocytes were activated and became permissive for HIV-1 replication when co-cultivated with cells expressing a functionally defective HIV-1 (F12/Hut-78 cells). This effect depended on the presence in F12/Hut-78 supernatants of nanovesicles we identified as exosomes. By inspecting the underlying mechanism, we found that ADAM17, i.e., a disintegrin and metalloprotease converting pro-TNF-α in its mature form, associated with exosomes from F12/Hut-78 cells, and played a key role in the HIV-1 replication in unstimulated CD4^+^ T lymphocytes. In fact, the treatment with an inhibitor of ADAM17 abolished both activation and HIV-1 replication in unstimulated CD4^+^ T lymphocytes. TNF-α appeared to be the downstream effector of ADAM17 since the treatment of unstimulated lymphocytes with antibodies against TNF-α or its receptors blocked the HIV-1 replication. Finally, we found that the expression of Nef_F12_ in exosome-producing cells was sufficient to induce the susceptibility to HIV-1 infection in unstimulated CD4^+^ T lymphocytes.

**Conclusions:**

Exosomes from cells expressing a functionally defective mutant can induce cell activation and HIV-1 susceptibility in unstimulated CD4^+^ T lymphocytes. This evidence highlights the relevance for AIDS pathogenesis of the expression of viral products from defective HIV-1 genomes.

## Background

The high frequency of mutations occurring in HIV-1 retrotranscription can lead to the production of defective HIV-1 genomes. They can persist in peripheral blood mononuclear cells (PBMCs) of HIV-1 infected patients since host cells are expected to have a lifetime longer than that of cells infected by replication-competent quasispecies. First evidences about the significant presence *in vivo* of defective HIV-1 genomes came from the observation that the number of PBMCs containing HIV-1 DNA greatly exceeds that of cells expressing infectious HIV-1 [1,2]. Later, 46% of HIV-1 genomes detected in PBMCs from 10 infected patients was found deleted, while PBMCs from 3 patients harbored only deleted or rearranged HIV-1 genomes [3]. Sequence analysis of the HIV-1 RT gene in PBMCs and rectal tissue of highly active anti-retroviral therapy (HAART)-treated patients revealed a great number of stop codons in all samples analyzed [4]. More recently, the analysis of 213 proviral clones from treated patients demonstrated the presence of 88.3% of genomes with identifiable defects [5].

Of major importance, mutations do not necessarily hamper the expression of defective HIV-1 genomes. Accordingly, defects in basically all HIV-1 genes except *tat* were identified in genomes of HIV-1 isolated from plasma of HAART-treated patients [6-10]. At least part of these mutated viral genomes are expected to integrate in host cell DNA thereby expressing defective HIV-1. Thus, the presence in HIV-1 infected patients, especially those treated by HAART, of defective but transcriptionally active HIV-1 genomes can be relevant, and investigating their role in the development of the disease would be of interest. We looked at the effects of the expression of a prototype of functionally defective HIV-1 (i.e., F12/HIV-1) [11] on bystander unstimulated CD4^+^ T lymphocytes. This system can mirror the events occurring *in vivo* upon interaction of resting lymphocytes with cells harboring defective HIV-1 genomes expressing either fully or partially functional viral products. The Hut-78 cells chronically infected with the non-producer F12/HIV-1 strain (referred to as F12/Hut-78 cells) were obtained by cloning cells infected by supernatants of PBMCs from an HIV-1 infected patient [11]. Cells expressing such HIV-1 mutant do not release infectious viral particles, meanwhile expressing a complete viral protein pattern comprising a truncated Vpr, an uncleaved Env gp160, and a mutated Nef (Table 1) [12]. In the present study, we provide evidence that exosomes released by F12/Hut-78 cells can influence the cell activation state of bystander, unstimulated CD4^+^ T lymphocytes.

**Table 1:** **Proteome of F12/HIV-1**^**a**^

***Viral gene***	***Amino acid substitutions***	***Viral protein product***
***gag***	p17: 1	Inefficient cleavage of the p55 precursor [12,13]
	p24: 6	
	p15: 3	
***pol***	Pro: 3	Apparently intact [12]
	RT: 10	
	Endo: 2	
***env***	gp120: 4	No cleavage of the gp160 precursor [11,12]
	gp41: 2	
***nef***	3	Defects in trafficking and signaling functions [13,14]
***tat***	0	Functional [12]
***rev***	0	Functional [12]
***vif***	14	Functionally defective [12,15]
***vpr***	3	Premature stop codon at aa 76 [12]
***vpu***	0	Apparently intact [12]

^a^The F12/HIV-1 amino acid sequence was compared to a consensus sequence obtained with multiple alignments of 5 type B HIV-1 isolates. No nucleotide substitutions have been found in the LTRs, TAR, and RRE HIV-1 regulatory sequences.

Exosomes are nanovesicles released by all cell types. They are lipid bilayer vesicles of 50–100 nanometers which form intracellularly upon inward invagination of endosome membranes [16]. This event leads to the formation of intraluminal vesicles which then become part of multivesicular bodies. Subsequently, they can undergo to lysosomal degradation or, alternatively, be released into extracellular space upon fusion of multivesicular bodies with plasma membrane. Exosomes can be released also through direct extrusion of plasma membrane [17]. Current protocols of purification and marker analysis cannot discriminate between endosome-produced nanovesicles and vesicles with similar size but originating from cell membranes. For the sake of clarity, cell-produced nanovesicles are here defined exosomes irrespectively to their origin.

It is now accepted that exosomes are part of the intercellular communication network while they were originally thought to secrete only waste cell material [18]. Exosomes incorporate messenger RNAs, microRNAs, and proteins which are functional in target cells [19]. Exosomes from HIV-1 infected cells incorporate Gag [20] and Nef HIV-1 proteins [21,22]. HIV-1 Gag molecules associate with exosomes by virtue of their higher-order oligomerization, while Nef is incorporated in exosomes upon anchoring into lipid raft microdomains through its N-terminal myristoylation and a stretch of basic amino acid residing in its alpha-helix 1.

Here, we demonstrate that the cell activation induced by exosomes from F12/Hut-78 cells in unstimulated human primary CD4^+^ T lymphocytes couples with HIV-1 replication. These effects relied on the expression of Nef_F12_.

## Results

### Unstimulated CD4^+^ T lymphocytes become susceptible to HIV-1 infection when co-cultivated with cells expressing a defective HIV-1 strain

**Figure 1:**
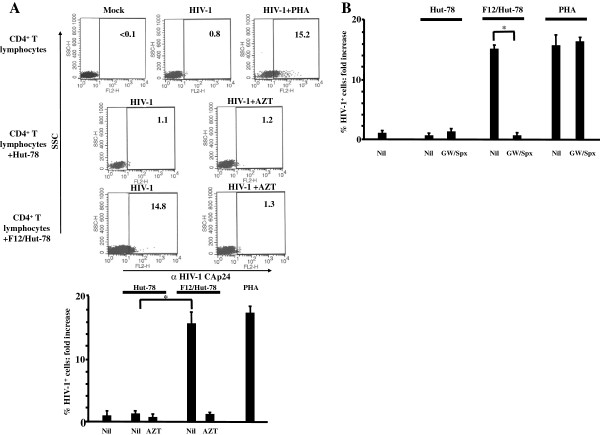
**Exosome-dependent HIV-1 replication in unstimulated CD4**^**+ **^**T lymphocytes after co-cultivation with Hut-78/F12 cells. A**. HIV-1 replicates in unstimulated CD4^+^ T lymphocytes co-cultured in *trans*-well plates with F12/Hut-78 cells. F12/Hut-78 cells or parental Hut-78 cells were put in the upper chamber of *trans*-well plates where unstimulated CD4^+^ T lymphocytes were seeded in the bottom chamber. After overnight co-culture, unstimulated CD4^+^ T lymphocytes were recovered and infected by a T-tropic HIV-1 strain in the presence or not of AZT. As control, unstimulated and PHA-treated CD4^+^ T lymphocytes were infected by HIV-1 or left untreated (Mock). Three days later, the lymphocyte cultures were analyzed by FACS for the expression of HIV-1 Gag-related products. In the upper displays, shown are the dot-plots of FACS analysis from a representative experiment. Percentages of HIV-1 Gag positive cells are indicated in each plot. In the bottom, the fold increases of the percentages of HIV-1 CAp24-positive cells compared to cells treated with HIV-1 alone are presented. Shown are the mean of fold increases + SD as calculated from three independent experiments with duplicates. *p < 0.05. **B**. Effects of the inhibitors of exosome release on the HIV-1 susceptibility of unstimulated CD4^+^ T lymphocytes co-cultured with F12/Hut-78 cells. *Trans*-well co-cultures were run as described for panel A, however in the presence or not of the inhibitors of exosome release GW4869 and Spiroepoxide. After overnight co-culture, the unstimulated CD4^+^ T lymphocyte cultures and, as control, PHA-activated lymphocytes treated or not with the inhibitors of exosome release were washed and infected by HIV-1. Three days later, the cell cultures were analyzed by FACS for the expression of HIV-1 Gag-related products. The fold increases of the percentages of HIV-1 CAp24-positive cells compared to cultures treated with HIV-1 alone are presented. Shown are the mean of fold increases + SD as calculated from three independent experiments with duplicates. *p < 0.05.

We looked at possible effects of the expression of defective HIV-1 on bystander unstimulated CD4^+^ T lymphocytes by setting up *trans*-well co-cultures of F12/Hut-78 cells with unstimulated CD4^+^ T lymphocytes isolated from healthy donors. F12/Hut-78 cells or, as control, parental uninfected Hut-78 cells were put in the upper chamber of a 0.4 μm *trans*-well plate where unstimulated CD4^+^ T lymphocytes were seeded in the bottom chamber. After overnight co-cultivation, unstimulated CD4^+^ T lymphocytes were recovered from *trans*-well plates and infected with HIV-1 in the presence or not of azidothymidine (AZT). Three days later, HIV-1 replication in unstimulated cells was evaluated by FACS analysis for the presence of intracytoplasmic HIV-1 Gag-related products. HIV-1 replicated in unstimulated CD4^+^ T lymphocytes from co-cultures with F12/Hut-78 cells but not parental ones (Figure 1A). The HIV-1 replication did not occur also in CD4^+^ T lymphocytes from co-cultures carried out in the presence of AZT (Figure 1A), thus ensuring that what we detected was consequence of authentic HIV-1 infection.

Considering that HIV-1 replication in lymphocytes needs cell activation, we hypothesized that the expression of the defective HIV-1 generates a signal leading to lymphocyte activation. To identify the nature of such a signal, we repeated the co-culture experiments in the presence of a number of drugs or antibodies. Among the drugs we tested, HIV-1 replication in bystander cells was successfully blocked by 1 μM of both GW4869 and Spiroepoxide (Figure 1B), i.e., two structurally unrelated inhibitors of neutral sphingomyelinase blocking exosome release [23-28]. This result prompted us to focus on exosomes as possible effectors of the induction of HIV-1 susceptibility in unstimulated CD4^+^ T lymphocytes.

### Characterization of exosomes from F12/Hut-78 cells

**Figure 2:**
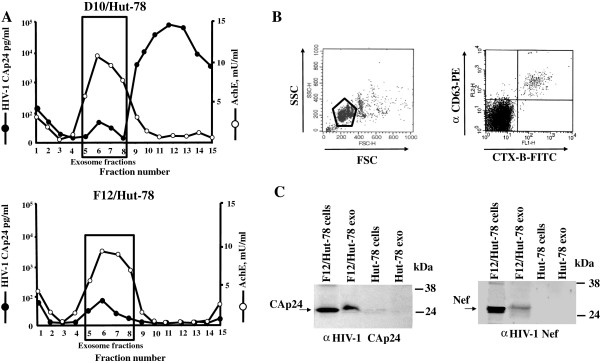
**Characterization of exosomes from supernatants of D10/Hut-78 and F12/Hut-78 cells. A**. HIV-1 Gag contents and AchE activity measured in fractions from 6-18% iodixanol gradients loaded with vesicles obtained by differential centrifugations of supernatants from D10/Hut-78 and F12/Hut-78 cells. The results are the mean values of duplicate conditions, and are representative of two independent experiments. **B**. Detection by FACS of GM1 and CD63 on pools of AchE positive fractions from iodixanol gradients loaded with vesicles from F12/Hut-78 cells. Vesicles were bound to aldehyde/sulfate latex beads and then labeled with both FITC-CTX-B and PE-conjugated anti-CD63 monoclonal antibody. Both FSC/SCC (on the left) and fluorescence (on the right) dot plots are reported. The gate on the left panel includes both single and doublet beads considered for the fluorescence analysis. The results are representative of three independent experiments carried out on vesicles from three iodixanol gradient preparations. **C**. Detection of HIV-1 CAp24 (on the left) and Nef (on the right) by western blot analysis of both cells and exosomes from F12/Hut-78 and uninfected, parental Hut-78 cells. On the left of each panel, arrows indicate the specific signals, while on the right molecular markers are given in kDa. Results are representative of two (for CAp24 detection) and six (for Nef detection) independent experiments.

Exosomes from F12/Hut-78 cells were isolated by differential centrifugations and then purified by 6-18% gradients of iodixanol. As control, the same procedure was applied to supernatants from D10/Hut-78 cells, i.e., a cell line chronically infected with a producer HIV-1 strain [11]. Gradient fractions were assayed for the activity of acetylcholinesterase (AchE, a marker of exosomes) [29], and the presence of HIV-1 Gag products (Figure 2A). As previously described [30], 6-18% iodixanol gradients concentrated exosomes in fractions 5–8. Here, Gag products also were detectable (Figure 2A), likely consequence of their association with exosomes [20]. Conversely, in denser fractions, where viral particles are expected to float, Gag products remained undetectable in gradients loaded with nanovesicles from F12/Hut-78 cells, while they massively accumulated in gradients with nanovesicles from D10/Hut-78 cells. Therefore, supernatants from F12/Hut-78 cells appeared to be free of viral particles. Hence, still possible residual viral particles were expected to only minimally contaminate the exosome preparations from F12/Hut-78 cells obtained through differential centrifugations.

Nanovesicles from F12/Hut-78 cells were formally identified as exosomes by FACS analysis demonstrating the presence of both monosialotetrahexosylganglioside (GM1) [31,32], using FITC-conjugated cholera toxin fraction B (CTX-B), and CD63 (Figure 2B). In addition, western blot analysis revealed that, consistently with what previously described for exosomes from Gag- [20] or Nef-expressing cells [21,22], both HIV-1 CAp24 and Nef proteins associate with exosomes from F12/Hut-78 cells (Figure 2C).

### Primary unstimulated CD4^+^ T lymphocytes release both IL-2 and TNF-α in response to the treatment with exosomes from F12/Hut-78 cells

**Figure 3:**
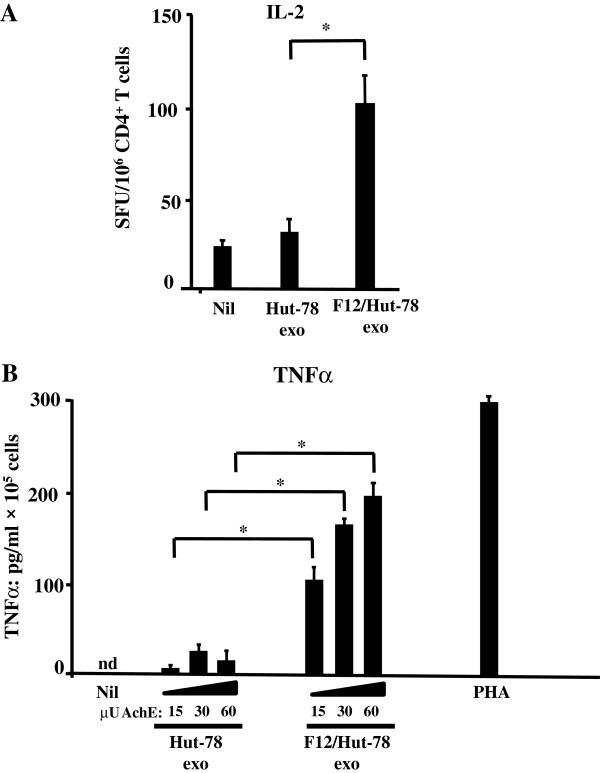
**Unstimulated CD4**^**+ **^**T lymphocytes release IL-2 and TNF-α when treated with exosomes from F12/Hut-78 cells. A**. Detection of IL-2-producing cells. 2 × 10^5^ unstimulated CD4^+^ T lymphocytes were challenged with 60 μU of exosomes from either Hut-78 or F12/Hut-78 cells, or mock-challenged (Nil), and then incubated for 48 hours in ELISPOT microwells previously coated with an anti-IL-2 monoclonal antibody. Shown are the mean number + SD of IL-2 spot forming units (SFU)/10^6^ cells calculated from five independent experiments. *p < 0.05. **B**. Detection of TNF-α on supernatants. 10^5^ unstimulated CD4^+^ T lymphocytes were challenged with increasing amounts (i.e., from 15 to 60 μU) of exosomes from Hut-78 or F12/Hut-78 cells, or mock-challenged (Nil). As control, cells were also treated with 2 μg/mL of PHA. After extensive washings, the cells were seeded in complete medium, and supernatants were harvested 6 hours later. TNF-α contents were determined by ELISA. Shown are the mean concentrations + SD as calculated from three independent experiments. Nd: not detectable. *p < 0.05.

HIV-1 replication in quiescent CD4^+^ T lymphocytes requires cell activation which associates with the release of interleukin (IL)-2 and tumor necrosis factor (TNF)α. We asked whether unstimulated CD4^+^ T lymphocytes release IL-2 and TNF-α in response to the treatment with exosomes from F12/Hut-78 cells. Human unstimulated CD4^+^ T lymphocytes were challenged with equal amounts of exosomes derived from F12/Hut-78 cells or, as control, uninfected Hut-78 cells. Exosomes were quantified in terms of the activity of AchE as detailed in the Material and Methods section. We assessed the number of IL-2 producing cells by ELISPOT assay 48 hours after exosome challenge, and the TNF-α release by ELISA on supernatants harvested at different hours after challenge. We found that 60 μU of exosomes from F12/Hut-78 cells applied on 10^5^ unstimulated CD4^+^ T lymphocytes cells generated a number of cells producing IL-2 significantly increased compared to untreated cells or cells treated with exosomes from parental Hut-78 cells (Figure 3A). Consistently, higher levels of TNF-α were detected when unstimulated CD4^+^ T lymphocytes were treated with scaled amounts (i.e., from 15 to 60 μU/10^5^ cells) of exosomes from F12/Hut-78 cells than from parental ones (Figure 3B). The TNF-α release peaked at 6 hours post challenge, and returned to control levels 16 hours after challenge (data not shown). No TNF-α was detected in the exosome preparations we used for the challenges (not shown).

These data implied that the treatment of unstimulated CD4^+^ T lymphocytes with exosomes from F12/Hut-78 cells leads to cell activation.

### The treatment with exosomes from F12/Hut-78 cells renders unstimulated CD4^+^ T lymphocytes susceptible to HIV-1 replication

**Figure 4:**
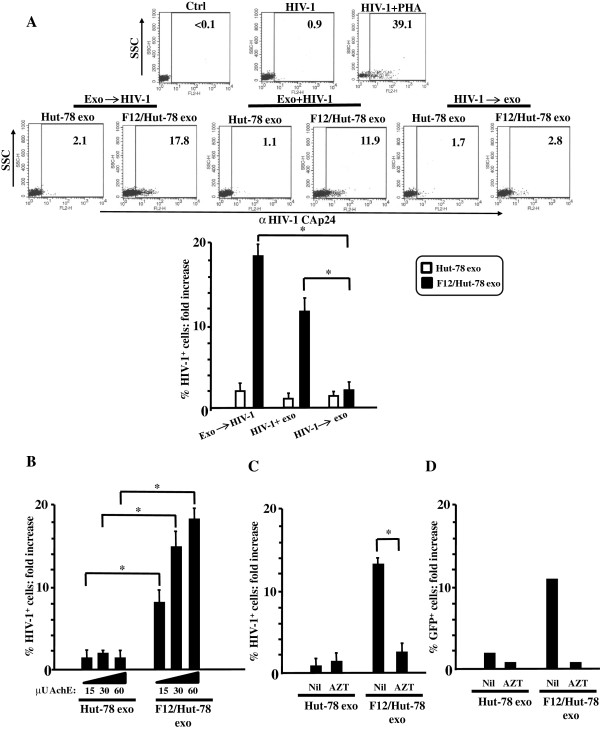
**HIV-1 replicates in unstimulated CD4**^**+ **^**T lymphocytes treated with exosomes from F12/Hut-78 cells. A**. FACS analysis of challenged lymphocytes. 10^5^ CD4^+^ T lymphocytes were challenged with 60 μU of exosomes from F12/HIV-1 cells, and then infected with HIV-1. Alternatively, exosomes and HIV-1 were added to cells at the same time, or cells were first infected and then challenged with the exosomes. As control, cells were infected with or without PHA. Three days later, the cells were scored for the expression of HIV-1 Gag by FACS analysis. In the upper panels, shown are the dot-plots from a representative experiment. Percentages of HIV-1 Gag positive cells are indicated. In the bottom panel, shown are the fold increases of the percentages of HIV-1 CAp24-positive cells compared to cultures treated with HIV-1 alone. Shown are the mean of fold increases + SD as calculated from five independent experiments with duplicates. *p < 0.05. **B**. Dose–response effect of exosomes. Shown are the mean of fold increases + SD as calculated from three independent experiments with triplicates. *p < 0.05. **C**. Effects of AZT. Cultures were run in the absence (Nil) or in the presence of 10 μM AZT. Shown are the mean of fold increases + SD as calculated from three independent experiments with duplicates. *p < 0.05. **D**. Detection of infectious HIV-1. 10^5^ CD4^+^ T lymphocytes were challenged with 60 μU of exosomes from either Hut-78 or F12/Hut-78 cells, and then infected with HIV-1 with or without AZT. Three days later, co-cultures with Rev-CEM cells were undertaken. After additional 3 days, GFP positive Rev-CEM cells were scored by FACS. Shown are the mean of fold increases of GFP^+^ Rev-CEM cells from exosome-treated cells compared to co-cultures with lymphocytes treated with HIV-1 alone, as calculated from two independent experiments with duplicates.

Next, we investigated whether the cellular activation induced by exosomes released from F12/Hut-78 cells couples with HIV-1 replication. As previously described [33], timing of quiescent CD4^+^ T lymphocyte activation strongly influences viral replication. Thus, exosomes were added to unstimulated CD4^+^ T lymphocytes either: i) six hours before infection with the T-tropic NL4-3 HIV-1 strain; ii) together with HIV-1, and iii) six hours after HIV-1 infection. HIV-1 replication was assayed by FACS analysis 3 days after infection by FACS analysis for the intracellular expression of HIV-1 CAp24. We measured 0.4 to 0.9% of HIV-1 CAp24 positive cells in unstimulated cells from different donors challenged with HIV-1 alone. Conversely, when exosome challenge preceded HIV-1 infection, a strong increase in the number of HIV-1 Gag expressing cells was observed (Figure 4A). The increase appeared slightly but reproducibly less striking when cells were simultaneously treated with exosomes and HIV-1, whereas negligible effects were observed when HIV-1 infection preceded exosome challenge (Figure 4A). On the basis of these results, the subsequent experiments were carried out by infecting cells with HIV-1 6 hours after exosome treatment.

Similar to what we observed for TNF-α release, the extents of HIV-1 replication in CD4^+^ T lymphocytes correlated with the exosome input (Figure 4B). Notably, the treatment with 10 μM AZT led to a strong reduction of HIV-1 CAp24-expressing CD4^+^ T lymphocytes (Figure 4C). This result formally excluded that possible carry-over from exosome- and/or virus-associated CAp24 interfered with the results we obtained by FACS analysis, meanwhile indicating that HIV-1 was indeed expressed in unstimulated CD4^+^ T lymphocytes challenged with exosomes from F12/Hut-78 cells.

Next, we were interested in establishing whether HIV-1 expressing CD4^+^ T lymphocytes released infectious virus. To this end, CD4^+^ T cells were challenged with exosomes from Hut-78 or F12/Hut-78 cells and then with HIV-1 in the presence or not of AZT. Three days later, CD4^+^ T cell cultures were washed, and co-cultures with Rev-CEM reporter cells, i.e., a cell line expressing GFP in the presence of both HIV-1 Tat and Rev products [34], were set up. After additional 3 days, the percentages of GFP-expressing Rev-CEM cells were evaluated by FACS (Figure 4D). No significant increase in the percentage of GFP^+^ cells was observed in Rev-CEM from co-cultures with CD4^+^ T lymphocytes treated with control exosomes, whereas a sharp increase was detectable when the lymphocytes were treated with exosomes from F12/Hut-78 cells. Such an increase was no more detectable when challenged CD4^+^ T lymphocytes were cultured in the presence of AZT until the addition of the reporter cells. These results indicated that unstimulated CD4^+^ T lymphocytes release infectious virus when treated with exosomes from F12/Hut-78 cells. In addition, the lack of infected Rev-CEM cells in the co-cultures with CD4^+^ T lymphocytes challenged with control exosomes indicated that HIV-1 replicating in Rev-CEM cells did not originate from the viral input used to infect the unstimulated CD4^+^ T lymphocytes.

We concluded that the exosome-dependent activation of CD4^+^ T lymphocytes rendered them susceptible to productive HIV-1 infection.

### ADAM17 is involved in both activation and HIV-1 replication in unstimulated CD4^+^ T lymphocytes treated with exosomes from F12/Hut-78 cells

**Figure 5:**
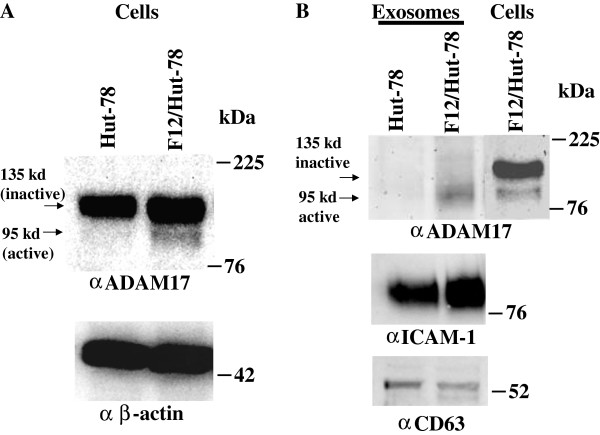
**Active ADAM17 associates with exosomes from F12/Hut-78 cells.** Western blot analysis for the expression of ADAM17 in both cells **(A)** and exosomes **(B)** from Hut-78 and F12/Hut-78 cells. On the left of blots for ADAM17, arrows identify both inactive and active ADAM17 forms. Signals from cellular ADAM17 were normalized with β-actin signals, while both anti-ICAM-1 and anti-CD63 monoclonal antibodies served to normalize ADAM17 signals from exosomes. On the right of each panel, molecular weight markers are given in kDa. Results are representative of five independent experiments.

Next, we investigated the mechanism underlying the cell activation in unstimulated CD4^+^ T lymphocytes targeted by exosomes from F12/Hut-78 cells. It has been recently reported that the expression of HIV-1 Nef leads to incorporation of active ADAM17 into exosomes. Upon ingestion of these exosomes, unstimulated CD4^+^ T lymphocytes release TNF-α as consequence of the cleavage of pro-TNF-α driven by exosome-associated ADAM17 [35]. ADAM17 belongs to the family of ADAM (*a d*isintegrin *a*nd *m*etalloprotease) enzymes [36]. It is a multi-domain, transmembrane, Zn^2+^-dependent proteinase whose inactive form presents a pro-domain which can be cleaved by furin in *trans*-Golgi network. The most characterized function of active ADAM17 is the cleavage of pro-TNF-α to its active form. We asked whether exosome-associated ADAM17 was involved in the CD4^+^ T lymphocyte activation we observed. To this aim, we first assayed the presence of active ADAM17 in exosomes from F12/Hut-78 cells. Western blot analysis highlighted the presence of active ADAM17 in F12/Hut-78 cells but not in parental Hut-78 cells (Figure 5A). Consistently, active ADAM17 was found in exosomes from F12/Hut-78 cells but not from uninfected cells (Figure 5B). Notably, inactive ADAM17 was not found in exosomes.

**Figure 6:**
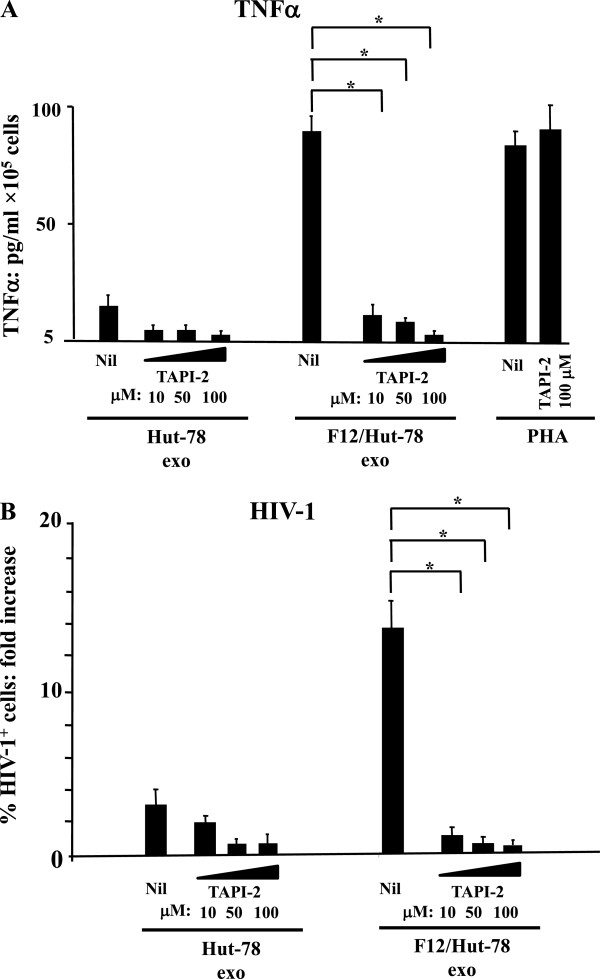
**The inhibition of ADAM17 activity impairs both TNF-α release and HIV-1 replication in unstimulated CD4**^**+ **^**T lymphocytes treated with exosomes from F12/Hut-78 cells. A**. Effect of TAPI-2 on TNF-α release. 10^5^ unstimulated CD4^+^ T lymphocytes were challenged with 60 μU of exosomes from either Hut-78 or F12/Hut-78 cells, or, as control, treated with 2 μg/mL of PHA. After extensive washings, the cells were cultured for 6 hours in the presence or not (Nil) of the indicated concentrations of TAPI-2. Thereafter, supernatants were harvested and titrated for the presence of TNF-α. Shown are the mean + SD of TNF-α concentrations as calculated from three independent experiments with duplicates. *p < 0.05. **B**. Effect of TAPI-2 on HIV-1 replication. 10^5^ unstimulated CD4^+^ T lymphocytes were challenged with 60 μU of exosomes from either Hut-78 or F12/Hut-78 cells, and then infected by HIV-1. As control, unstimulated cells were challenged with HIV-1 alone, or in the presence of 2 μg/mL of PHA. The cells were then left in culture for 3 days in the presence or not (Nil) of the indicated concentrations of TAPI-2. Finally, the cells were analyzed for HIV-1 expression by FACS analysis. The fold increases of the percentages of HIV-1 CAp24-positive cells compared to cultures treated with HIV-1 alone are reported. Shown are the mean of fold increases + SD as calculated from three independent experiments with duplicates. *p < 0.05.

To test a possible contribution of ADAM17 shuttled by exosomes, we treated CD4^+^ T lymphocytes with TAPI-2, i.e., a potent ADAM17 inhibitor [37]. We observed that TAPI-2 inhibited the release of TNF-α from unstimulated CD4^+^ T lymphocytes challenged with exosomes from F12/Hut-78 cells (Figure 6A). Consistently, TAPI-2 also inhibited the viral replication in unstimulated CD4^+^ T lymphocytes challenged with exosomes from F12/Hut-78 cells and then infected with HIV-1 (Figure 6B). Taken together, these data highlight the role that exosome-associated ADAM17 plays in the activation and HIV-1 replication in CD4^+^ T lymphocytes treated with exosomes from F12/Hut-78 cells.

### Both TNF-α neutralization and blocking TNF-α receptors-1 and -2 inhibit the HIV-1 replication in primary unstimulated CD4^+^ T lymphocytes treated with exosomes from F12/Hut-78 cells

**Figure 7:**
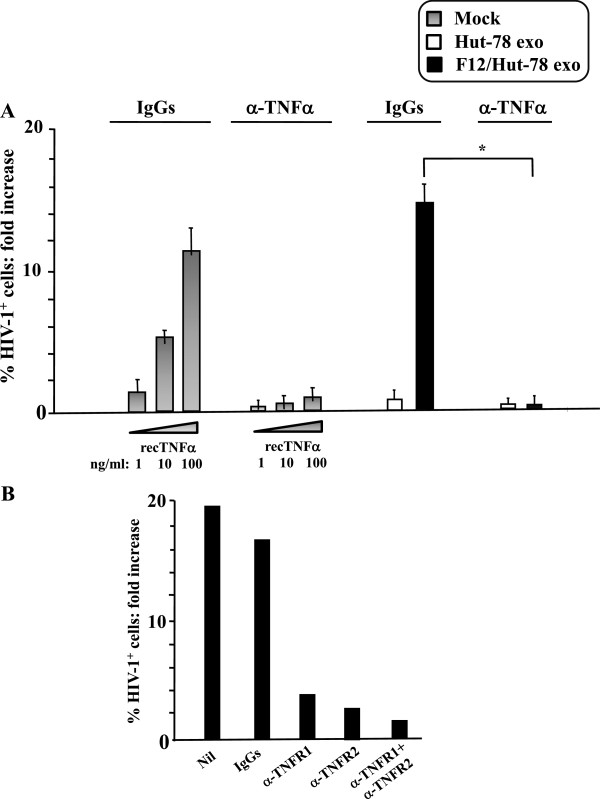
**TNF-α drives the HIV-1 replication in unstimulated CD4**^**+ **^**T lymphocytes treated with exosomes from F12/Hut-78 cells. A**. Neutralization of soluble TNF-α. 10^5^ unstimulated CD4^+^ T lymphocytes were challenged with 60 μU of exosomes from either Hut-78 or F12/Hut-78 cells, and then infected with HIV-1. After washings, the cells were cultured in the presence of anti-TNF-α neutralizing antibodies or with unrelated IgGs. As control, unstimulated CD4^+^ T lymphocytes were treated with the indicated doses of recombinant TNF-α, infected by HIV-1, and then cultured in the presence of anti-TNF-α neutralizing antibodies or IgGs. Three days after challenges, the cells were analyzed for HIV-1 expression by FACS analysis. The fold increases of the percentages of HIV-1 CAp24-positive cells compared to cultures treated with HIV-1 alone are presented. Shown are the mean of fold increases + SD as calculated from three independent experiments with duplicates. *p < 0.05. **B**. Effects of the block of TNFR1 and TNFR2. The same procedure described for panel A was applied, except than unstimulated lymphocytes were incubated with neutralizing anti-TNFR1 or -TNFR2 antibodies either alone or in combination, or irrelevant isotype IgGs for 1 hour at 4°C before exosome and HIV-1 challenges. Three days later, the cells were analyzed for HIV-1 expression by FACS analysis. The fold increases of the percentages of HIV-1 CAp24-positive cells compared to lymphocytes treated with HIV-1 alone are presented. Shown are the mean of fold increases as calculated from two independent experiments with duplicates.

Besides ADAM17, TAPI-2 inhibits other ADAMs, ACE secretase, and other metalloproteinases. To ensure that the TAPI-2-dependent inhibitory effects correlated with inhibition of ADAM17, we next investigated the role of the major ADAM17 substrate, i.e., TNF-α, in our experimental setting. Unstimulated CD4^+^ T lymphocytes were challenged with exosomes and HIV-1, and then cultured for 3 days in the presence of anti-TNF-α neutralizing antibodies. For control, cells challenged with HIV-1 alone were treated with recombinant TNF-α in the presence/absence of the anti-TNF-α antibodies. We observed that anti-TNF-α antibodies totally abolished HIV-1 replication in CD4^+^ T lymphocytes treated with exosomes from F12/Hut-78 cells as well as in cells treated with recombinant TNF-α, which, as expected, increased HIV-1 replication in a dose-dependent manner (Figure 7A).

To further strengthen the role of TNF-α, experiments with neutralizing antibodies against the TNF-α receptors (TNFR)-1 and 2, either alone or in combination, were performed. According to what we observed with anti-TNF-α antibodies, the treatment with anti-TNFR1 and –TNFR2 antibodies alone or in combination inhibited the HIV-1 replication in unstimulated CD4^+^ T lymphocytes challenged with exosomes from F12/Hut-78 cells (Figure 7B). This result suggests that the activation of both TNFR1- and TNFR2-related intracellular signals is required for the HIV-1 replication in unstimulated CD4^+^ T lymphocytes.

Overall, these results supported the data we obtained with TAPI-2, and are consistent with the idea that TNF-α was the downstream effector of exosome-associated ADAM17.

### Unstimulated CD4^+^ T lymphocytes replicate HIV-1 when targeted by exosomes released by cells expressing Nef_F12_

**Figure 8:**
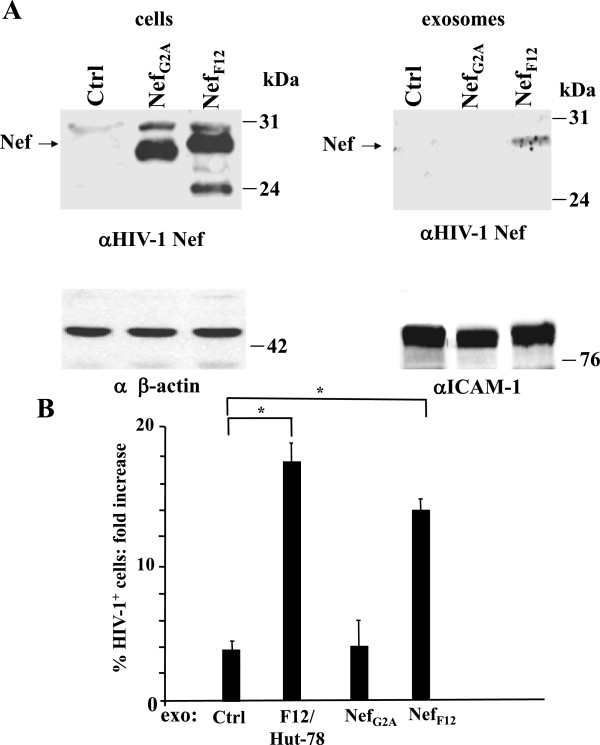
**HIV-1 replicates in unstimulated CD4**^**+ **^**T lymphocytes treated with exosomes from Nef**_**F12**_**-expressing cells. A**. Detection of Nef in cells expressing either Nef_F12_ or Nef_G2A_, and in exosomes purified from the respective supernatants. Shown is the anti-Nef western blot analysis of both cells and exosomes from either 293 T cells transiently transfected with vectors expressing Nef_F12_ or Nef_G2A_, or mock-transfected cells (Ctrl). Signals from cellular Nef were normalized with β-actin detection, and anti-ICAM-1 analysis served to normalize exosome signals. On the left of Nef-specific panels, arrows indicate the specific signals. On the right, molecular markers are given in kDa. Results are representative of two independent experiments. **B**. HIV-1 replication in unstimulated CD4^+^ T lymphocytes treated with exosomes from cells expressing either Nef_F12_ or Nef_G2A_. 10^5^ unstimulated CD4^+^ T lymphocytes were challenged with 60 μU of exosomes from either F12/Hut-78 cells, 293 T cells transfected with Nef_F12_- or Nef_G2A_-expressing vectors, or mock-transfected cells (Ctrl), and then infected with 50 ng of HIV-1. As control, unstimulated cells were challenged with HIV-1 alone. Three days later, the cells were analyzed by FACS for HIV-1 expression. The fold increases of the percentages of HIV-1 CAp24-positive cells compared to cultures treated with HIV-1 alone are reported. Shown are the mean of fold increases + SD as calculated from three independent experiments with duplicates. *p < 0.05.

Active ADAM17 was previously reported to be uploaded in exosomes as consequence of the ectopic expression of wild-type Nef in producer cells [35]. The upload of active ADAM17 in exosomes from F12/Hut-78 cells was suggestive of a key role of Nef_F12_ in the induction of HIV-1 susceptibility in CD4^+^ T lymphocytes. To investigate this point, 293 T cells were transiently transfected with vectors expressing Nef_F12_ or, as control, Nef_G2A_, i.e., a mutant which is not expected to assemble the Nef-associated kinase complex required for ADAM17 uploading in exosomes [38]. This phenotype is the consequence of the lack of the N-terminal myristoylation site which dramatically decreases the efficiency of Nef association with cell membranes. We noticed that Nef_F12_, differently to Nef_G2A_, was incorporated into exosomes (Figure 8A). Unstimulated CD4^+^ T lymphocytes were challenged with equal amounts of exosomes derived from cells expressing either Nef_F12_ or Nef_G2A_. As control, cells were treated with exosomes derived from either mock-transfected cells or F12/Hut-78 cells. Six hours later, the cells were infected with HIV-1, and after additional 3 days, analyzed for HIV-1 expression. HIV-1 replicated at similar extents in cell cultures treated with exosomes from F12/Hut-78 cells or Nef_F12_-expressing cells, while no HIV-1 replication was detectable in cells treated with control or Nef_G2A_ exosomes (Figure 8B).

These results indicate that the expression of Nef_F12_ in exosome-producing cells is sufficient to render unstimulated CD4^+^ T lymphocytes susceptible to HIV-1 infection.

### Unstimulated CD4^+^ T lymphocytes become susceptible to HIV-1 infection when co-cultivated with activated CD4^+^ T lymphocytes infected by a Nef_F12_-expressing, non-producer HIV-1

**Figure 9:**
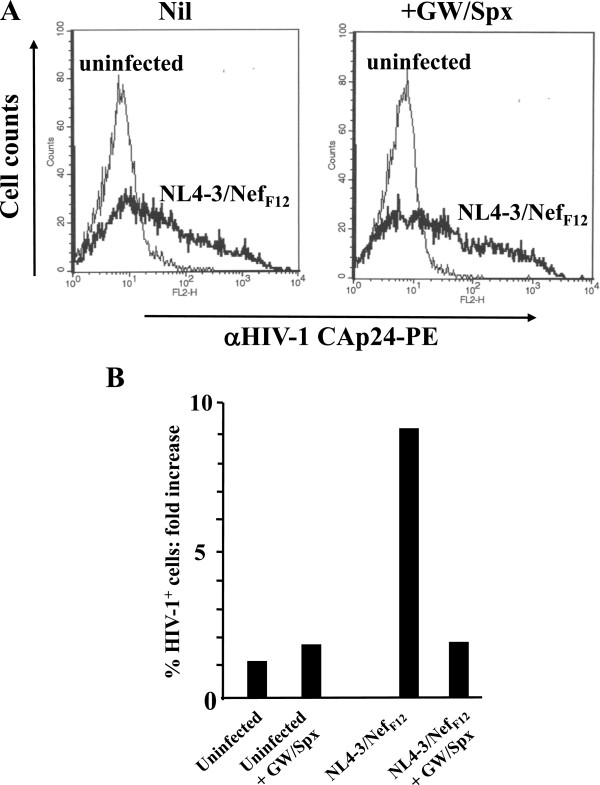
**HIV-1 replicates in unstimulated CD4**^**+ **^**T lymphocytes co-cultured in *trans*-well plates with activated CD4**^**+ **^**T lymphocytes expressing the non-producer NL4-3/Nef**_**F12 **_**HIV-1 strain.** CD4^+^ T lymphocytes were activated by PHA and, 2 days later, either infected by 100 ng CAp24/10^6^ cells of (VSV-G) NL4-3/Nef_F12_ HIV-1, or mock infected. After 2 additional days, the infected cells were put in the upper chamber of *trans*-well plates where unstimulated CD4^+^ T lymphocytes were seeded in the bottom chamber. After overnight co-culture in the presence or not of GW4869 and Spiroepoxide, the unstimulated CD4^+^ T lymphocytes were recovered and infected by a T-tropic HIV-1 strain. As control, at the same time untreated CD4^+^ T lymphocytes were infected by HIV-1. Three days later, the lymphocyte cultures were analyzed by FACS for the HIV-1 expression. **A**. CAp24 FACS analysis of PHA-activated CD4^+^ T lymphocytes infected with (VSV-G) NL4-3/Nef_F12_ HIV-1 at the time of recovery of unstimulated CD4^+^ T lymphocytes from *trans*-well chambers. The fluorescence plots are overlaid with those from untreated or treated uninfected cells. **B**. Fold increases of the percentages of HIV-1 CAp24-positive unstimulated CD4^+^ T lymphocytes from the co-cultures 3 days after the challenge with HIV-1. The HIV-1 positive percentages were compared to those measured in HIV-1 infected, untreated cells. Shown are the mean of fold increases as calculated from two independent experiments with duplicates.

Finally, we were interested in establishing whether Nef_F12_ expressed in both viral and cellular contexts different from F12/Hut-78 cells maintains its key role in inducing HIV-1 susceptibility on bystander CD4^+^ T lymphocytes. To this end, PHA-activated primary CD4^+^ T lymphocytes were infected by a NL4-3 HIV-1 strain where wt Nef was replaced by Nef_F12_[39], and which was pseudotyped with the G protein from vesicular stomatitis virus (VSV-G). As for F12/Hut-78 cells, cells expressing this HIV-1 strain do not release viral particles, in the presence however of the expression of a complete viral protein pattern [39]. Both mock and infected cell cultures were put in the upper chamber of *trans*-well plates where unstimulated CD4^+^ T lymphocytes were seeded in the lower one, in the presence or not of the exosome inhibitors GW4869 and Spiroepoxide. After an overnight incubation, unstimulated CD4^+^ T lymphocytes were recovered from *trans*-well plates and infected with HIV-1. Three days later, HIV-1 replication in unstimulated cells was evaluated by FACS analysis. We noticed that HIV-1 replicated in unstimulated CD4^+^ T lymphocytes from co-cultures with cells infected with the NL4-3/Nef_F12_ HIV-1 strain but not mock infected cells (Figure 9). The viral replication was no more detectable when the co-cultures were carried out in the presence of the exosome inhibitors (Figure 9).

These results add significance to what we previously observed with 293 T transfected cells in terms of the key role of Nef_F12_ expression in the HIV-1 replication in bystander CD4^+^ T lymphocytes.

## Discussion

Formal demonstration of the presence of grossly defective HIV-1 genomes in PBMCs of AIDS patients was achieved in mid ‘90s [3]. Physical mapping of defective genomes indicated that the frequency of deletions is proportional to their proximity to the central part of the viral genome [3]. Subsequent studies demonstrated the presence of genetically damaged sequences in transcriptionally active proviral HIV-1 [40]. Moreover, HAART treatment further selects for mutated/deleted viral genomes in both PBMCs and rectal tissues [4,41]. The identification of an *in vitro* cell system comprehensively reproducing the events which *in vivo* lead to the formation of defective HIV-1 DNA is still an unmet challenge. The functionally defective F12/HIV-1 genome [42] could recapitulate some features of different defective HIV-1 species which *in vivo* remain transcriptionally active.

Our investigations started from the observation that unstimulated CD4^+^ T lymphocytes became susceptible to HIV-1 infection upon *trans*-well co-culture with F12/Hut-78 cells. This experimental setting was useful to avoid misinterpretations due to possible effects induced by cell-to-cell contact. However, it is fairly conceivable that it mirrors events occurring in free co-cultures and, more important, in tissues most relevant for HIV replication, e.g., gut mucosa and lymph nodes.

Unstimulated CD4^+^ T lymphocytes challenged by exosomes from F12/Hut-78 cells released both IL-2 and TNF-α. The fact that no IL-2 production was detectable until 36–48 hours after exosome challenge (data not shown) suggested that its release was consequence of the cellular activation induced by an autocrine/paracrine stimulus of TNF-α. The prompt release of TNF-α appears to be the key event leading to the activation of unstimulated CD4^+^ T lymphocytes challenged by exosomes from F12/Hut-78 cells.

It was reported that HIV-1 does not replicate in quiescent CD4^+^ T lymphocytes due to its inability to complete retrotranscription and integration steps [33,43,44]. On the other hand, TNF-α has manifold effects on HIV infection: for instance, it activates resting CD4^+^ T lymphocytes meanwhile stimulating TAK, i.e. a cell kinase required for the Tat *trans*-activation [45]. It can also re-activate latent HIV-1 through the action of its downstream effectors [46]. Our evidences that unstimulated CD4^+^ T lymphocytes became susceptible to HIV-1 infection when the exosome-mediated stimulus was applied before but not after HIV-1 infection are highly reminiscent of previously published data on resting CD4^+^ T lymphocytes stimulated with anti-CD3/CD28 antibodies before or after HIV-1 challenge [33]. In this experimental setting, a great increase of both fully retrotranscribed and integrated products was found in pre-stimulated compared to post- and non-stimulated cells. On the basis of these literature data, we hypothesize that the TNF-α released upon exosome challenge helps the viral genome to complete early replication events. Upon provirus integration, the activation of the downstream effectors of both TNF-α and IL-2 would contribute to foster HIV-1 transcription.

We found active ADAM17 in F12/Hut-78 cells and exosomes released by them, but not in uninfected cells. Despite the cells accumulate much higher amounts of inactive ADAM17 compared to the active form, only the latter seemed to be uploaded in exosomes. The mechanism of selective upload of active ADAM17 deserves further investigations.

We cannot formally exclude that Nef_F12_ delivered in target cells by exosomes could contribute to the cell activation/HIV-1 replication in unstimulated lymphocytes. However, the fact that Nef_F12_ allele lacks many Nef signaling functions [13,14] runs against this hypothesis. Most important, the domain critical for ADAM17 activation and uploading into exosomes (i.e., the N-terminal 11–40 region) [35,38] is conversely well conserved in Nef_F12_[12].

Our results allow to propose a model where cells expressing defective HIV-1 genomes release ADAM17-loaded exosomes which, when ingested by bystander quiescent CD4^+^ T lymphocytes, can induce the cleavage of pre-stored pro-TNF-α. The mature cytokine may act in an autocrine/paracrine way by activating intracellular signals supporting the progression of the life cycle of infectious HIV-1. Thus, our results suggest that defective HIV-1 genomes which are transcriptionally active could play a relevant role in supporting the spread of replication-competent HIV. Furthermore, they strengthen previous evidences about the key role of TNF-α in HIV pathogenesis [47].

This mechanism would be of particular importance in HAART-treated patients where defective HIV-1 genomes accumulate in the presence of the block of the replication of infectious virus. In this scenario, the activation of resting CD4^+^ T lymphocytes induced by ADAM17-uploaded exosomes may foster the spread of replication-competent latent HIV, which was estimated to represent 11.7% of genomes [5], as well as the emergence of drug-resistant HIV quasispecies and, when the therapy is interrupted, infectious HIV. In addition, a sustained production of exosomes inducing the release of active TNF-α could contribute to the overall immune activation still present in successfully HAART-treated patients.

## Conclusions

Exosomes released by cells expressing a defective HIV-1 genome activate bystander, resting CD4^+^ T lymphocytes and render them susceptible to HIV-1 infection. Exosome-associated ADAM17 plays a key role in both cell activation and HIV-1 replication observed in CD4^+^ T lymphocytes, and TNF-α is its downstream effector. Overall, our data reveal a so far neglected effect of defective but transcriptionally active HIV-1 genomes on bystander lymphocytes which could play a relevant role in the spread of HIV-1 in infected persons.

## Methods

### Cell cultures and isolation

Hut-78, D10/Hut-78, F12/Hut-78 [11], and Rev-CEM cells [34] were grown in RPMI medium plus 10% heat-inactivated fetal calf serum (FCS). Human embryonic kidney 293 T cells were grown in Dulbecco‘s modified Eagle’s medium plus 10% heat-inactivated fetal calf serum (FCS). CD4^+^ T lymphocytes were isolated from PBMCs of healthy donors by negative selection using an immunomagnetic-based kit (Miltenyi), and cultivated in RPMI medium plus 10% FCS. The cell cultures were checked for their purity through FACS analysis for CD4, CD8, and CD14 markers. Cell preparations having more than 3% of CD8^+^ cells and /or 1% of CD14^+^ cells were discarded. For activation, 2 μg/mL of phytohemagglutinin (PHA) were added to CD4^+^ T lymphocyte cultures. TAPI-2 was purchased from Santa Cruz Biotechnology. Recombinant human TNF-α was from R&D Systems. AZT was obtained from NIH AIDS Research and Reference Reagent Program. For anti-TNF-α neutralization experiments, 1 μg of either anti-TNF-α neutralizing antibodies (polyclonal rabbit antibodies, Fitzgerald Industries) or normal rabbit IgGs was added to CD4^+^ T lymphocyte immediately after exosome and HIV-1 challenges. The same amounts of antibodies were then re-added after 24 hours of culture. For TNFR-blocking experiments, anti-TNFR1 clone #16085 and anti-TNFR2 clone #22210 neutralizing monoclonal antibodies (both from R&D Systems) were used. CD4^+^ T lymphocytes were incubated for 1 hour at 4°C with 1 μg of the antibodies either alone or in combination or, as control, with the same amount of isotype control IgGs, and then challenged with exosomes and HIV-1. Antibodies were then re-added 24 hours later.

### Molecular clones, transfections, and HIV-1 infections

Preparations of T-tropic HIV-1 were obtained from the supernatants of 293 T cells 48 hours after transfection with the pNL4-3 HIV-1 molecular clone. The VSV-G pseudotyped NL4-3/Nef_F12_ HIV-1 strain [39] was obtained by co-transfecting the HIV-1 molecular clone with a pcDNA3.1 (Invitrogen)-based vector expressing the VSV-G in a 5:1 molar ratio. Both Nef_F12_ and Nef_G2A_ alleles were cloned in the pcDNA3.1 vector after PCR amplification from the HIV-1 molecular clones expressing the respective Nef mutant [12,48]. Transfections were performed using Lipofectamine 2000 (Invitrogen). Supernatants were clarified and concentrated by ultracentrifugation on a 20% sucrose cushion as previously described [49]. This method ensured that exosomes from transfected cells were excluded from the vesicle pellet [50]. Virus preparations were titrated in terms of HIV-1 CAp24 content using quantitative enzyme-linked immunosorbent assay (ELISA, Innogenetic). Infections with HIV-1 were carried out by spinoculation at 400 × *g* for 30 min at room temperature (r.t.) using 500 ng CAp24 equivalent of HIV-1 for 10^6^ cells.

### *Trans*-well co-cultures

*Trans*-well co-cultures were carried out in 12-well plates using Cell Culture Insert Falcon Membrane (25 mm diameter, 0.4 μm pore size, Becton Dickinson). They were set up by putting 10^6^ F12/Hut-78 or parental Hut-78 cells in the upper chamber, while 2 × 10^6^ unstimulated CD4^+^ T lymphocytes were seeded in the bottom chamber. The co-cultures were then run overnight in the presence or not of 1 μM of the inhibitors of exosome release GW4869 (Sigma) and Spiroepoxide (Santa Cruz). Then, CD4^+^ T lymphocytes were recovered and infected with HIV-1, washed, and left in culture 3 days in the presence or not of 10 μM AZT. Afterwards, lymphocytes were analyzed by FACS for the HIV-1 expression through the detection of intracytoplasmic HIV-1 CAp24.

### Nanovesicle purification and challenge

Cell culture supernatants containing exosome-depleted FCS were processed following already described methods for exosome purification. In detail, supernatants were centrifuged at 500 × *g* for 10 min and filtered with 0.22 μM pore size. Then, the supernatants underwent differential centrifugations consisting in a first ultracentrifugation at 10,000 × *g* for 30 min. Supernatants were then harvested and ultracentrifuged at 70,000 × *g* for l h. The pelleted vesicles were resuspended in 1 × PBS, and ultracentrifuged again at 70,000 × *g* for 1 h. Afterwards, the pellet was resuspended in 200 to 400 μL of 1 × PBS and, in some cases, subjected to discontinuous iodixanol (Axis-Shield) gradient. It was performed essentially as described [51]. Briefly, concentrated vesicles were ultracentrifuged at 200,000 *×* g for 1.5 hours at 4°C in an SW41 Ti rotor (Beckman) through a 6 to 18% iodixanol density gradient formed by layering iodixanol in 1.2% increments. Then, 0.7 ml fractions were collected starting from the top. In some instances, half of each fraction was diluted with 2 volumes of 0.9% sodium chloride and ultracentrifuged for 30 min at 95,000 rpm in a TL-100 tabletop ultracentrifuge. Finally, the pellet was resuspended in 50 μl of Tris–HCl pH 7.4 10 mM, NaCl 100 mM, EDTA 1 mM, 0.1% Triton X-100.

### AchE activity assay

The vesicle-associated AchE activity was evaluated through the Amplex Red kit (Molecular Probes) following the manufacturer’s recommendations. The AchE activity was measured as mU/mL, where 1 mU is defined as the amount of enzyme which hydrolyzes 1 pmole of acetylcholine to choline and acetate per minute at pH 8.0 at 37°C.

### FACS analysis of cells and nanovesicles

For the detection of intracytoplasmic HIV-1 CAp24, cells were treated with trypsin for 15 min at 37°C. Then, they were labeled using the KC57-RD anti-CAp24 monoclonal antibody (Coulter) upon permeabilization with Cytofix/Cytoperm solutions (BD Pharmingen) as previously described [52].

Double staining of nanovesicles was performed by incubating them with 5 μl of surfactant-free white aldehyde/sulfate latex beads overnight at r.t. on a rotating plate. The binding of exosomes with the beads was necessary for both antibody labeling and FACS analysis. Afterwards, nanovesicle-bead complexes were washed and incubated at 37°C for 2 hours with FITC-conjugated CTX-B. Then, the samples were washed and incubated with PE-conjugated anti-CD63 monoclonal antibody (BD Pharmingen) 1 h at 37°C. Finally, the beads were washed, resuspended in 1 *×* PBS-2% v/v formaldehyde, and FACS analyzed.

### Western blot assay

Western blot analysis on cell lysates was performed by washing cells twice with 1 × PBS (pH 7.4) and lysing them for 20 min on ice with lysis buffer (20 mM HEPES pH 7.9, 50 mM NaCl, 10 mM EDTA, 2 mM EGTA, 0.5% nonionic detergent IGEPAL CA-630, 0.5 mM dithiothreitol, 20 mM sodium molybdate, 10 mM sodium orthovanadate, 100 mM sodium fluoride, 10 μg/mL leupeptin, 0.5 mM phenylmethylsulfonyl fluoride). Whole cell lysates were centrifuged at 6,000 *× g* for 10 min at 4°C. The protein concentration of cell extracts was determined by the Lowry protein quantitation assay. Aliquots of cell extracts containing 30 to 50 μg of total proteins were resolved by 8-12% sodium dodecyl sulfate-polyacrylamide gel electrophoresis (SDS-PAGE) and transferred by electroblotting on a 0.45 μm pore size nitrocellulose membrane (Amersham) overnight using a Bio-Rad Trans-Blot. For western blot analysis of exosomes, they were lysed and analyzed as described for cell lysates. For immunoassays, membranes were blocked with 5% non-fat dry milk in PBS containing 0.1% Triton X-100 for 1 hour at room temperature, then incubated overnight at 4°C with specific antibodies diluted in PBS containing 0.1% Triton X-100. The antibodies used in immunoblots were: polyclonal rabbit anti-HIV-1 Gag CAp24 #4250 (NIH AIDS Research and Reference Program), sheep polyclonal anti-Nef ARP444 (a generous gift from Dr. Mark Harris), rabbit polyclonal anti-ADAM17 from Cell Signaling, monoclonal anti-ICAM-1 15.2 from Santa Cruz Biotech., monoclonal anti-CD63 from R&D Systems, and monoclonal anti-β-actin AC-74 (Sigma). Immune complexes were detected with horseradish peroxidase conjugated goat anti-rabbit or goat anti-mouse antibodies (GE Healthcare) and enhanced chemiluminescence reaction (Euroclone).

### IL-2 and TNF-α detection

The release from CD4^+^ T lymphocytes of IL-2 and TNF-α was detected by ELISPOT and ELISA, respectively. For IL-2, cells were treated with exosomes and then cultivated for 48–72 hours in ELISPOT microwells (Millipore) previously coated with a monoclonal antibody against human IL-2 (Mabtech). Afterwards, the cells were removed, and a biotinylated antibody against human IL-2 was added, followed by the addition of a streptavidin-alkaline phosphatase. The plate was finally developed using BCIP/NBT substrate (Sigma). Spot-forming cells were analyzed and counted using an ELISPOT reader (Amplimedical Bioline A-EL-VIS GmbH). The measurement of TNF-α was performed through ELISA kits from Immunological Sciences following the manufacturer’s recommendations.

### Statistical analysis

When appropriate, data are presented as mean + standard deviation (SD). In some instances, the paired Student’s *t*-Test was used and confirmed using the non-parametric Wilcoxon rank sum test. *P* < 0.05 was considered significant.

## Competing interests

The authors declare that they have no competing interests.

## Authors’ contributions

CA carried out the most part of cell and infection experiments. CC carried out ELISA, ELISPOT, FACS, and western blot analyses. SCC produced and purified exosomes, and carried out AchE-based quantification assays of exosomes. FM performed blocking experiments with anti-TNFR antibodies and virus titrations using Rev-CEM cells. MF supervised the research and wrote the manuscript. All authors read and approved the final manuscript.
